# Central catheter-related Gordonia bronchialis bacteremia in an immunocompromised patient: A case report, and literature review

**DOI:** 10.1016/j.idcr.2023.e01738

**Published:** 2023-03-06

**Authors:** Mohammed Alnajjar, Deena Mudawi, Honar Cherif, Samar Mahmoud Hashim, Ahmed Zaqout, Amina Bougaila, Farah Imadeldden Jibril, Shehab Fareed Mohamed

**Affiliations:** a Division of Internal Medicine, Hamad Medical Corporation, Doha, Qatar; b Division of Hematology, National Center for Cancer Care and Research (NCCCR), Doha, Qatar; c Division of Infectious Diseases, Department of Medicine, Hamad Medical Corporation, Doha, Qatar; d Communicable Disease Center, Hamad Medical Corporation, Doha, Qatar; e Division of Pharmacy, NCCCR, Doha, Qatar

**Keywords:** Gordonia, Central line, Bacteremia, AML

## Abstract

Gordonia is a rarely reported organism causing central line-associated bloodstream infection (CLABSI). This article reports an acute myeloid leukemia (AML) case in which the patient developed febrile neutropenia and was later found to have Gordonia bronchialis (G. bronchialis) CLABSI. The patient received a two-week ceftriaxone regimen, based on susceptibility. The microbiologic diagnosis of this organism is considered challenging due to its resemblance with other organisms; however, more sophisticated methods of diagnosis (such as gene sequencing) can aid in differentiation.

## Introduction

Gordonia is a genus of gram-positive bacteria that belongs to the family Gordoniaceae, which was previously classified in the Rhodococcus genus [1], [2]. It is rarely reported as a human pathogen in local and systemic diseases. The latter is more commonly described in immunocompromised cases, particularly those with indwelling catheters [3], [4]. Furthermore, it poses a diagnostic challenge due to its prolonged incubation time and the need for advanced microbiological investigations to be detected [5]. This article reports a case of central line infection caused by Gordonia in an immunocompromised patient with acute myeloid leukemia (AML). In addition, we reviewed the literature for the previously reported cases of Gordonia-related line infection and summarized the findings in the Table 1.

**Table 1:** Line-related Gordonia bacteremia cases.

Case	Age/Gender	Specie	Comorbid	Indwelling	Time to positive culture
Ramanan et al., 2013[12]	38/M	*Otiditis*	AML	Hickman	20 hr
	48/M	*Polyisoprenivorans*	AML	Hickman	32 hr
	4/F	*N/A*	ALL	Hickman	24–48 hr
Renvoise et al., 2009[13]	69/M	*Sputi*	Prostate CA, DM, HTN, Alcohol	Central line	N/A
Lesens et al., 2000[14]	31/F	*Sputi*	Thalassemia, Cirrhosis	PICC	N/A
Verma et al., 2006[15]	78/M	*polyisoprenivorans*	MDS	Hickman	24 hr
Pham et al., 2003[16]	28/M	*terrae*	CML	Central line	N/A
Buchman et al., 1992[17]	44/F	*terrae*	Brain tumor	Central line	N/A
	54/F	*terrae*	AML	Central line	N/A
	46/F	*terrae*	Metastatic liver cancer	Central line	N/A
	60/M	*terrae*	Thyroid CA	Central line	N/A
Villanueva et al., 2016[11]	85/M	*Rubropertincta*	ESRD	Hickman	8 days
	91/M	*Sputi*	ESRD	Hickman	N/A
Grisold et al., 2007[18]	24/M	*Terrae*	HCM	Central line	4 days
Kofteridis et al., 2012[19]	70/F	*Sputi*	Breast CA	Hickman	N/A
Kempf et al., 2004[20]	26/F	*Polyisoprenivorans*	CML, BMT	Hickman	3 days
McCormick et al., 2022[21]	56/F	*bronchialis*	Burkitt lymphoma	Port-a-cath	N/A

M: Male; F: Female; AML: Acute Myeloid Leukemia; ALL: Acute Lymphoblastic Leukemia/Lymphoma; CA: Cancer; DM: Diabetes Mellitus; HTN: Hypertension; MDS: Myelodysplastic Syndrome; CML: Chronic Myelogenous Leukemia; ESRD: End Stage Renal Disease; HCM: Hypertrophic Cardiomyopathy; BMT: Bone Marrow Transplant; hr: hours; N/A: Not available

## Case report

**Fig. 1:**
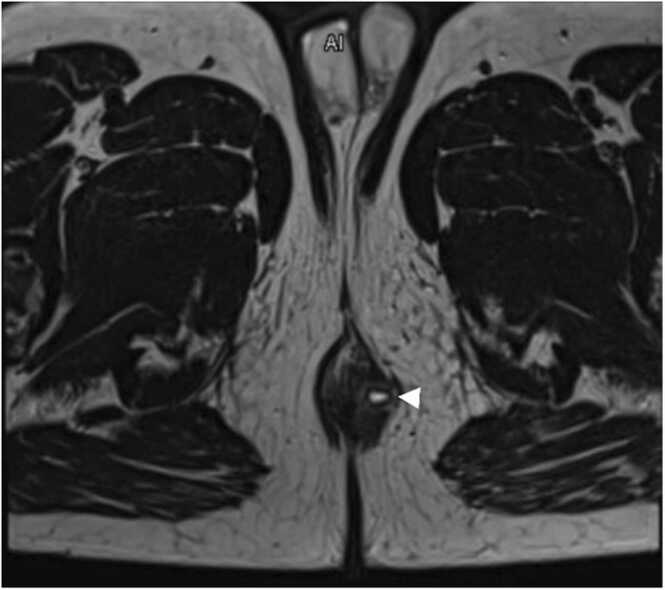
T2 weighted MRI image of the perineum area: Shows hyperintense lesion (arrowhead) on the left side of anal canal, suggestive of left-sided perianal fistula.

A 46-year-old gentleman with a past medical history of AML was admitted to our hospital as a case of AML relapse for salvage chemotherapy. During his hospital stay, the patient developed febrile neutropenia. On physical examination found to have perianal swelling, which was tender with overlying red skin. Magnetic Resonance Imaging (MRI) of the pelvis was done, which showed an underlying perianal collection of 0.4 × 0.8 × 1.8 cm; that developed left-sided perianal fistula with cutaneous communication (with the lower third of the anal canal at 4:00 o′clock), traversing the external anal sphincter (Fig. 1).

In the beginning, the patient was treated empirically with meropenem and tigecycline. Later, blood culture results were positive for Extended Spectrum Beta Lactamase (ESBL) Klebsiella pneumoniae organism, and tigecycline was discontinued while the patient was kept on meropenem. Meanwhile, the patient's condition improved clinically as he had no more fever spikes, and laboratory test results showed a decrease in inflammatory markers (C-Reactive protein from 95 to 8 mg/dL, Procalcitonin 2.08–0.35 ng/mL). Later, the patient underwent incision and drainage of the perianal abscess due to persistent perianal pain. The drained pus was sent for culture and showed mixed growth of anaerobic organisms.

Ten days after drainage, the patient developed another spike of high-grade fever (Temperature 39.4 C oral). At that time, the patient had no complaints. A full septic workup was conducted, including blood cultures from both peripheral and central lines taken simultaneously, and the patient was restarted on tigecycline. A complete blood count (CBC) test showed improvement in his absolute neutrophil count (ANC) to 0.8 × 10^3^ cells/mm^3^ (nadir during admission reached zero). After over three days from the last fever spike, an aerobic blood culture from a peripherally inserted central catheter (PICC) line grew G. bronchialis; However, peripheral blood cultures (one aerobic and one anerobic) did not grow any organism. Transthoracic Echocardiogram was done and demonstrated no valvular vegetations.

With this positive culture, it was decided to remove his PICC line. Susceptibility tests showed that the identified organism was resistant to trimethoprim-sulfamethoxazole but was sensitive to all other tested antibiotics: amoxicillin-clavulanate, ciprofloxacin, and clarithromycin. The patient was then started on ceftriaxone, based on susceptibility. The patient received 14 days of a two-gram daily dose of ceftriaxone. He was doing very well after that, with no relapse, and is currently planning a bone marrow transplant (BMT) from a related donor.

## Discussion

Gordonia organism is a weak acid-fast bacterium rod-shaped with cord-like colony formation, and it was first identified in the sputum of diseased pulmonary cases, particularly those with bronchiectasis or lung cavities. This type of bacteria has mixed characteristics of both Mycobacterium and Nocardia [6], [7]. Gordonia organisms are known for their ability to form a biofilm called gordonan, an acidic polysaccharide that induces cell aggregation, increases their pathogenicity and ability to cause bacteremia, particularly in patients with indwelling devices, such as central lines, peritoneal dialysis catheters, and cardiac devices [8].

The genus includes 28 species; however, only 9 species have been reported to cause infections in humans [7], [9]. The systemic disease caused by Gordonia is described mainly in immunocompromised patients, such as those with malignancy, diabetes, cardiovascular disease, and autoimmune conditions [5].

The lack of a precise diagnostic pathway, the prolonged incubation period of three to four days, and the need for thorough biochemical and morphological identification tests, make the diagnosis of Gordonia challenging. This organism can be identified using advanced non-phenotypic methods of identification, such as 16 S ribosomal RNA (rRNA) gene sequencing or matrix-assisted laser desorption/ionization-time of flight mass spectrometry (MALDI-TOF MS) [5], [9].

In one antibiotics susceptibility study involving 31 strains of Gordonia, all strains showed sensitivity to aminoglycosides (such as tobramycin, amikacin, and gentamicin). Besides, ampicillin was effective in all cases except in three strains: one bronchialis and two sputi [9]. Currently, there are no clear guidelines for the duration of antibiotics to cover this organism. In one case series, all pediatric patients used a four-to-six-week period of antibiotics and showed good outcomes; however, a two-week duration of both meropenem and gentamicin was used for one subject, later the patient developed a recurrence of infection [10]. Villanueva et al. used a two-week regimen of meropenem for one patient; however, the patient was re-admitted after two weeks and found to be infected with the same organism. The other case showed persistent bacteremia even after three weeks of proper antibiotics [11].

## Conclusion

Gordonia is a rare, emerging human microorganism that causes a wide range of infectious diseases, particularly debilitating sepsis in cancer patients. Blood cultures that show gram-positive bacilli are often overlooked as potential diphtheroid contaminants. Phenotypic methods are not sufficient for detecting these pathogens, and it is essential to use more sophisticated genotypic identification methods. By increasing awareness of Gordonia species, clinical practice among physicians and microbiologists will improve, leading to better survival outcomes for patients.

Further multi-center studies are necessary to identify the organism's antibiograms and to guide clinicians towards evidence-based guidelines for the appropriate duration of treatment.

## Ethical approval

Ethical approval was taken from Medical Research Center (MRC) Qatar before submitting this manuscript.

## Funding sources

The publication of this manuscript is supported by Qatar National Library (QNL).

## Consent

Written informed consent was obtained from the patient to publish this case report and any accompanying images.

## CRediT authorship contribution statement

Mohammed Alnajjar: Manuscript writing, literature review. Deena Mudawi, Honar Cherif, Samar Mahmoud Hashim, Ahmed Zaqout, Amina Bougaila, Farah imadeldden Jibril: Case identification, literature review, revisions in manuscript. Shehab Fareed Mohamed: Manuscript writing, literature review, review, and approval of the final manuscript.

## Conflict of interest

All authors involved declared no potential conflicts of interest.

## Data Availability

No datasets were generated or analyzed during the current study.
